# Empowering Privacy Through Peer-Supervised Self-Sovereign Identity: Integrating Zero-Knowledge Proofs, Blockchain Oversight, and Peer Review Mechanism

**DOI:** 10.3390/s24248136

**Published:** 2024-12-20

**Authors:** Junliang Liu, Zhiyao Liang, Qiuyun Lyu

**Affiliations:** 1 School of Computer Science and Engineering, Macau University of Science and Technology, Macau, China; 2 School of Cyberspace, Hangzhou Dianzi University, Hangzhou 310005, China

**Keywords:** data security, privacy protection, self-sovereign identity (SSI), blockchain, zero-knowledge proof, chameleon hash

## Abstract

Frequent user data breaches and misuse incidents highlight the flaws in current identity management systems. This study proposes a blockchain-based, peer-supervised self-sovereign identity (SSI) generation and privacy protection technology. Our approach creates unique digital identities on the blockchain, enabling secure cross-domain recognition and data sharing and satisfying the essential users’ requirements for SSI. Compared to existing SSI solutions, our approach has the practical advantages of less implementation cost, ease of users’ understanding and agreement, and better possibility of being soon adopted by current society and legal systems. The key innovative technical features include (1) using a zero-knowledge proof technology to ensure data remain “usable but invisible”, mitigating data breach risks; (2) introducing a peer review mechanism among service providers to prevent excessive data requests and misuse; and (3) implementing a comprehensive multi-party supervision system to audit all involved parties and prevent misconduct.

## 1. Introduction

In recent years, incidents of user data leakage have become increasingly frequent both domestically and internationally. In 2018, Aadhaar, India’s national identity authentication system, leaked sensitive data of 1.1 billion Indian citizens. Similarly, Starwood Hotels exposed 500 million consumer records, and Facebook faced a data breach affecting 87 million users.

In 2020, 7.4 billion records were leaked from various sources, including the French newspaper Le Figaro. That same year, 538 million pieces of Weibo data were sold on the darknet, and employees of China Construction Bank illicitly sold over 50,000 pieces of information [1,2,3].

In 2021, on June 14, cybersecurity firm Cognyte leaked 5 billion pieces of sensitive information [4]. More recently, in 2024, AT&T experienced a data breach that impacted nearly all its customers and many non-customers.

These incidents involve three types of entities: users, service providers, and regulatory agencies, each facing distinct challenges and concerns. Service providers require user data to deliver services, leading to the accumulation of large amounts of user data, which creates data silos and increases the risk of data breaches [5]. On the other hand, users must provide their data to service providers without the needed control over these entities, exposing them to data breaches and misuse risks.

Second, illegitimate gainers see the demand for data and the opportunity for illegal profit. They can obtain information about many users directly (internal personnel) or indirectly (cyber attackers). Therefore, they will continue to steal and leak user information.

For regulatory agencies, regulation is often reactive, meaning regulators cannot prevent incidents from occurring. Attackers exploit this issue by targeting various service providers extensively. When a service provider is breached, a substantial amount of data, often stored in plain text, is compromised.

In summary, the issues associated with these user data security incidents can be distilled into four key points:

- User Data Breaches: Service providers often tend to store user data in plain text, making each provider a potential single point of failure.
- User Data Misuse: Users lack control over the data collected by service providers, which increases the risk of unauthorized use of these data.
- Excessive Data Collection: Service providers often collect more user data than necessary, heightening the risks of data breaches and misuse.
- Insufficient Regulation: Regulatory measures are typically reactive and lack stringent constraints on the behaviors of both users and service providers.

Based on the four problems above, the following solutions are desired:

1. User Control Over Personal Data: Users should have greater control over their data.
2. Encryption of User Data: Storing and using user data in an encrypted format rather than plain text can significantly reduce risks.
3. Unified User Authentication and Data Sharing: Implementing a standardized method for user authentication and data sharing would allow service providers to share data across domains, reducing the need for separate storage.
4. Peer Review Mechanism: Introducing an effective peer review mechanism can ensure that no single service provider collects more user data than necessary for the industry, addressing the issue of excessive data collection.
5. Enhanced Regulatory Measures: Strengthening regulatory methods to ensure mutual monitoring among all interaction parties can address the lack of regulation.

Our research is designed to realize these solutions. Our approach not only introduces self-sovereign identity (SSI) to ensure that users have control over the data they provide to service providers but also guarantees that the relevant data can be utilized across domains in a standardized form and can be accepted by multiple service providers (SPs) without the need for data transformation.

The innovative features of this research that made critical contributions to the quality of the proposed system are listed below.

1. Incorporating zero-knowledge proofs (ZKPs) for privacy protection in SSI mechanisms: ZKPs enhance the perpetual privacy of information during multiparty computations, ensuring that information remains “usable but invisible” during its flow and usage. Our approach uniquely brings ZKP, safeguarding user data in scenarios where plain text interaction is unnecessary.
2. Development of a blockchain-based content-sharing oversight mechanism: Existing SSI generation mechanisms often lack regulatory frameworks to mitigate malicious behavior by users and service providers. Our research introduces an innovative three-party supervisory mechanism, empowering regulatory bodies to effectively trace and monitor all behaviors of parties involved in information sharing on the blockchain. This comprehensive oversight system ensures no blind spots in regulatory coverage and holds all interacting parties accountable, including the regulatory bodies.
3. Introduction of a peer review mechanism to prevent excessive data requests: Current solutions often overlook the risk of service providers excessively requesting user data. Our research introduces a peer review mechanism that evaluates the necessity of service providers’ requests for data. This mechanism, supported by regulatory authorities, ensures that any additional data requests undergo scrutiny by industry peers, thereby mitigating the risk of data misuse.

The paper is structured as follows: Section 2 introduces and discusses the status of user identity and data usage, including a survey of the existing development processes of user identity, along with a comparative analysis of the advantages and disadvantages of the latest practices related to self-sovereign identity. Section 3 introduces the proposed system. Section 4 introduces the experimental analysis of the system, including formal verification using Scyther, theoretical analysis of specific key concerns through logical reasoning, and simulation experiments. Section 5 provides a summary and a forward-looking perspective on the future of our research.

## 2. Related Work

ISO/IEC 24760-1 defines identity as “a set of attributes associated with an entity” [6]. In digital identity, this “set of attributes” refers to the information computer systems used to represent external entities, including individuals, organizations, applications, or devices. When the entity in question is a person, digital identity often contains elements related to citizenship and personal identity. A digital identity encompasses the entire collection of information generated by a person’s online activities, such as usernames, passwords, online search history, date of birth, social identification, and personal privacy information [7].

Given this definition, user data fall under the category of digital identity and are suitable for management within a digital identity framework. Current models for managing digital identity include centralized, federated, user-centric, and self-sovereign identities.

### 2.1. Centralized Identity

Centralized identity manages and controls users’ digital identities by a single institution or organization. In the early days of the Internet, centralized institutions became the issuers and certifiers of digital identities, and most service providers still use this model. The essence of the centralized identity system is that the central organization holds the identity information, as the authentication and authorization of the information are also determined by this centralized organization [8]. Consequently, identity is not controlled by the user. Additionally, the problem is that different centralized websites have different identity systems, where the corresponding account information used is not interoperable across websites, forcing users to register dozens of identities on various sites without controlling any of them. This problem becomes more severe as the users deal with an increasing number of websites.

### 2.2. Federated Identity

Federated identities are managed and controlled by multiple federal agencies, first proposed by Microsoft’s Passport [9] project, which envisages federated identities that allow users to use the same identity on various websites. In other words, compared with the one-to-one correspondence between user digital identities and service providers in centralized identities, in federated identities, users can obtain a digital identity from a federation and use this identity to identify multiple service providers [10]. The mechanism of federated identities improves the Balkanization problem of centralized identities. However, the result is still an oligarchy, where the centralized powers of centralized identities are now dispersed among several powerful entities, and users still have no control.

### 2.3. User-Centric Identity

The user-centric identity mechanism eliminates the need for a federation, allowing digital identities to be managed across multiple individuals or institutions. It assumes that users can create and maintain a persistent online identity (ID) on their own, which can be used to interact with various service providers [11]. Many digital identity projects are based on this concept, including OpenID [12], OpenID 2.0 [13], OpenID Connect [14], OAuth [15], and FIDO [16].

User-centric approaches focus on two main elements: user empowerment and interoperability. These approaches allow users to authorize and share their identities from one service to another. For instance, OpenID enables users to register their own OpenID, allowing them to access multiple service providers without needing to register again. However, the technical complexity often leads most Internet users to prefer obtaining an OpenID from a long-term, reliable website. While this approach offers users autonomy and control over their identities, it also introduces a dependency on the specific website providing the OpenID. If the OpenID is managed by an external website rather than the user, the actual autonomy of the user is compromised, making this mechanism resemble a centralized identity model.

### 2.4. Self-Sovereign Identity

Self-sovereign identity (SSI) represents the next stage in the evolution of user-centric identity management, where the user is at the center of identity management [17]. SSI ensures the interoperability of user identities across multiple platforms with user consent and accurate user control over their digital identities, creating genuine user autonomy. This means users can manage and control their identity data, deciding when, how, and with whom to share their information.

The core principles of SSI include decentralization, autonomy, and privacy protection. For an SSI to achieve these goals, it must be portable and not tied to a specific site or region. Blockchain technology is a suitable choice to meet these requirements. Current SSI systems in the industry include Sovrin [18,19,20] and ShoCard [21]. Sovrin is dedicated to standardizing self-sovereign identity (SSI) and creating the necessary infrastructure, utilizing blockchain to store distributed identity. In theory, anyone can issue or verify an identity. The SSI model Sovrin employs does not rely on any specific distributed ledger and can work with any blockchain that meets the required attributes. All private data are stored off-chain by each self-sovereign identity owner, who decides where to keep it. However, this setup also allows users with insufficient security awareness to share plaintext information. Many may not fully grasp the implications of sharing unencrypted data or the importance of protecting their digital identity. This gap in understanding can lead to unintentional privacy breaches. Sovrin relies on legal frameworks like GDPR to restrict the misuse of information, which is only sometimes practical. Under significant pressure from technological and regulatory constraints, Sovrin is increasingly struggling to operate and may cease operations on 31 March 2025 [22].

ShoCard is a blockchain-based digital identity and verification platform. Users’ identity information is stored on the blockchain as signed encrypted hashes. On the blockchain, users initiate identity verification handshakes with third parties. The information is in secure data envelopes that only the recipient can decrypt. This method allows users and third-party entities to securely and verifiably confirm each other’s identities and share additional data. However, it does not fully address the issue of data misuse. While the shared data are transmitted in encrypted form, third parties can ultimately decrypt it to access plaintext information. This subsequent use of the data is beyond the user’s control, leading to significant privacy concerns. ShoCard has been rebranded as PingOne and is operating commercially; however, the similar design between the two still raises related security issues [23].

uPort is an open identity system that allows users to register their digital identity, known as uPortID, on the Ethereum blockchain [24]. Users can control the creation of their uPortID and decide how to share their personal information with third parties. Personal data are stored on the user’s device and in off-chain areas designated by the user, ensuring they always have access to it. uPort places greater control and responsibility for the uPortID in the hands of the user [25,26]. However, like Sovrin, uPort cannot protect user privacy through its protocol. If users mistakenly provide plaintext information, it can lead to privacy risks. Additionally, uPort does not address the legitimacy of third-party requests for user data or the management of its use, which may result in users being excessively asked for unnecessary information and experiencing data misuse.

Veramo is a new iterative version of uPort at a developmental level and continues to evolve [27]. It provides an open-source library with modular APIs for self-sovereign identity (SSI) and verifiable data [28,29]. However, the related issues persist due to the similarities in design between the two.

TCID is a self-sovereign identity system designed by Quinten Stokkink and colleagues in collaboration with the Dutch government [30]. TCID posits that without addressing privacy issues at the network level, SSI systems cannot fulfill their privacy protection promises. TCID includes components, including a communication layer and two peer-to-peer network overlay layers, designed to meet seven functional requirements that ensure ideal system attributes. While TCID emphasizes and optimizes privacy and performance at the network level, it does not delve deeply into potential issues within SSI, such as data misuse, excessive data requests, or the lack of effective oversight.

DT-SSIM provides a decentralized, trusted self-sovereign identity management framework for Internet of Things (IoT) environments [31]. This framework aims to enhance the security and decentralization of identity management in IoT by combining secret sharing and blockchain technology. By distributing the storage of identity credentials, the DT-SSIM framework strengthens the protection of these credentials and reduces the risks associated with centralized storage. Although decentralizing storage mitigates issues related to data monopolies, DT-SSIM still lacks controls to address challenges in SSI, such as data misuse, privacy breaches, excessive data requests, and limited oversight from data recipients.

PT-SSIM, an updated version of DT-SSIM, introduces a frequent identity share update mechanism, enhancing the protection of users’ digital identities and reducing the likelihood of data collection, linkage attacks, aggregation, and inference attacks [32]. However, it remains insufficient in tackling core issues of privacy breaches, misuse, and lack of oversight.

SISSI is a self-sovereign identity (SSI)-based network authentication and authorization system designed for semantic interoperability across different SSI approaches [33]. The SISSI architecture demonstrates how existing Web-based SSI technologies can support access control with enhanced end-to-end performance over blockchain-based methods. While SISSI enables cross-domain recognition among SSI systems and effective integration via Web methods, it lacks exploration of privacy issues, such as data misuse and oversight challenges. Furthermore, entirely replacing blockchain with Web approaches may increase the risk of centralization.

INCHAIN is an innovative network insurance architecture that leverages blockchain technology to ensure data transparency and traceability, automates the insurance process through smart contracts, and strengthens identification using self-sovereign identity (SSI) [34]. It significantly advances combating fraudulent claims and ensuring client identity verification. INCHAIN focuses on identifying and detecting claim initiators for insurers but overlooks the risk of malicious behavior among interacting entities within SSI. A design with mutual oversight among parties would be more effective. Additionally, architecture lacks measures to prevent potential data misuse or excessive data requests by insurers, posing privacy risks for claim initiators.

AASSI balances privacy and accountability with two modes: Normal Mode, allowing users to generate credentials for access control, and Accountability Mode, where trackers can trace real identities in collaboration with issuers [35]. While AASSI provides effective regulation by concealing identities in typical interactions and revealing them to designated trackers, it risks potential tracker misuse. Mutual oversight might enhance security, and the system lacks safeguards against data leaks, misuse, and excessive data requests within SSI.

## 3. Methodology

Our solution divides the process into four stages: registration, usage, update, and deletion of digital identity. Interactions between four types of entities and four types of blockchains are applied in one or more stages using multiple smart contract functions. The names and descriptions of the entities are shown in Table 1, and the names and descriptions of the blockchains are shown in Table 2. The symbols and their descriptions used in this Section are provided in Table 3. We will introduce the usage of various smart contract functions separately in each of the four stages of the solution.

### 3.1. Stage 1: Digital Identity Registration Stage

In the digital identity registration stage, users initially register and create their digital identity. This stage involves six steps and utilizes two smart contract functions. The names and descriptions of the smart contract functions are shown in Table 4.

**Figure 1:**
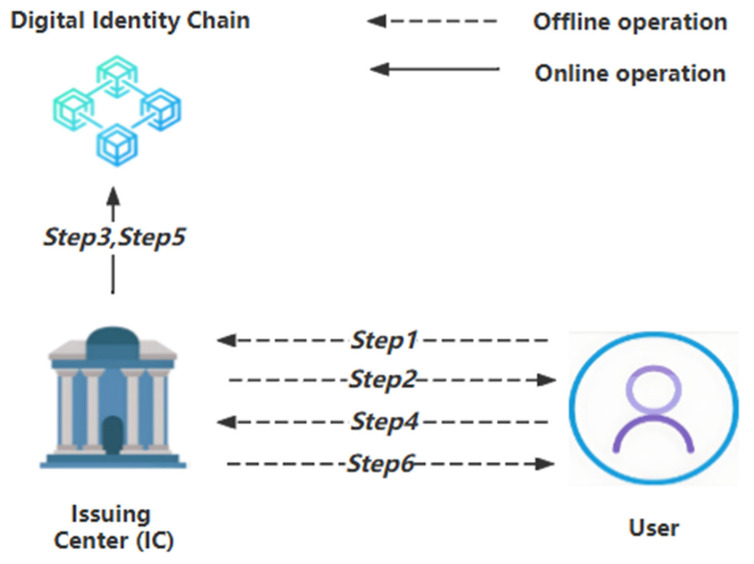
Interaction steps of digital identity registration stage.

The interaction steps of this stage are illustrated in Figure 1.

Step 1: The user goes offline to the Issuing Center (IC), providing their physical proof of identity and the public key PubU to apply for and obtain a digital identity. We can describe the process with the following symbolic description:

User → IC: {Physical Identity, PubU}(1)

Step 2: The Issuing Center (IC) verifies the user’s physical identity and, upon confirming its authenticity, provides them with a UID, which will serve as the unique identifier for the user’s digital identity. We can describe the process with the following symbolic description:

IC → User: {UID}(2)

Step 3: The Issuing Center (IC) creates a new user digital identity block on the digital identity chain using the smart contract function Create(PriIC(UID, PubU)), whereas PriIC is the private key of the Issuing Center. We can describe the process with the following symbolic description:

IC → Digital Identity Chain: {Create(PriIC(UID, PubU))}(3)

Step 4: The user provides a set of personal information {PI_id, PI_phone, … PI_n} for registration. All this information is real and in plaintext. We can describe the process with the following symbolic description:

User → IC: {PI_id, PI_phone, … PI_n}(4)

Step 5: The Issuing Center (IC) physically verifies the authenticity of the set of personal information {PI_id, PI_phone, … PI_n} that the user wishes to register, corresponding to physical characteristics. Upon successful verification, IC and the user jointly generate a set of personal information verification {PIV_id, PIV_phone, … PIV_n}. Each PIV_x item is a set containing {PriIC(PriU(Hash1(PI_x·Nonce))), PriIC(PriU(Hash2(PI_x·Nonce))), …, PriIC(PriU(Hashm(PI_x·Nonce)))}. IC updates the above content to the chain by a call of the smart contract Add(PriIC(UID, Hash(PubSC(PIV_x), PIN_x, m), Ts), PubSC(PIV_x), PIN_x, m). PubSC is the public key of the smart contract, and all smart contract functions use the same public–private key pair in our design. Although mainstream blockchains have not widely adopted cryptographic operations within smart contracts, there are methods in the industry to achieve our goals. For example, the enterprise blockchain platform Hyperledger Fabric supports encryption and decryption operations within chain code [40]. Alternatively, off-chain services can be used to perform encryption and decryption operations. Smart contracts can communicate with these external services to handle cryptographic tasks. For instance, decentralized oracles like Chainlink can securely connect smart contracts with external cryptographic services [41].

We can describe the process with the following symbolic description:

IC → Digital Identity Chain: {Add(PriIC(UID, Hash(PubSC(PIV_id), PIN_id, m), Ts), PubSC(PIV_id), PIN_id, m) … Add(PriIC(UID, Hash(PubSC(PIV_n), PIN_n, m), Ts), PubSC(PIV_n), PIN_n, m)}(5)

Step 6: The Issuing Center (IC) gives the user the random number Nonce generated in Step 5. The user must securely store this random number, which will be used for subsequent verification steps in the digital identity usage stage. We can describe the process with the following symbolic description:

IC → User: {Nonce}(6)

**Figure 2:**
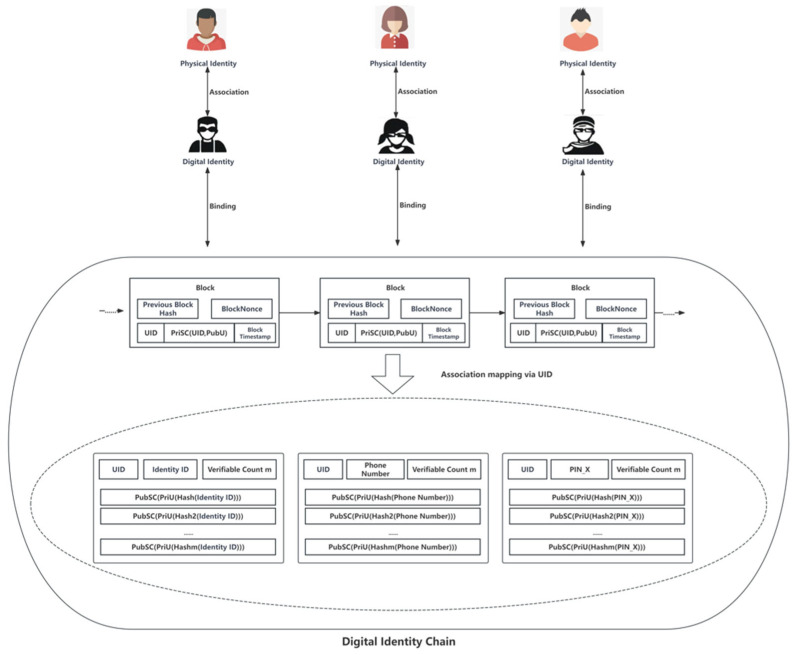
User’s association with their digital identity (idealized view).

Upon completion of the digital identity registration stage, the user’s association with their digital identity is illustrated in Figure 2. It is important to note that this is an idealized view provided for ease of understanding. The branching chain design shown in the diagram will not exist in actual storage. Instead, data will be stored linearly and accessed through smart contracts.

### 3.2. Stage 2: Digital Identity Usage Stage

In the digital identity usage stage, users will utilize their registered digital identities to interact with service providers (SPs) to obtain specific services. This stage will involve two scenarios: sharing UID only and sharing the UID along with specific categories of user personal information. We will discuss each scenario separately. This stage will utilize three smart contract functions; their names and descriptions are shown in Table 5.

#### 3.2.1. Scenario 1: Sharing UID Only

**Figure 3:**
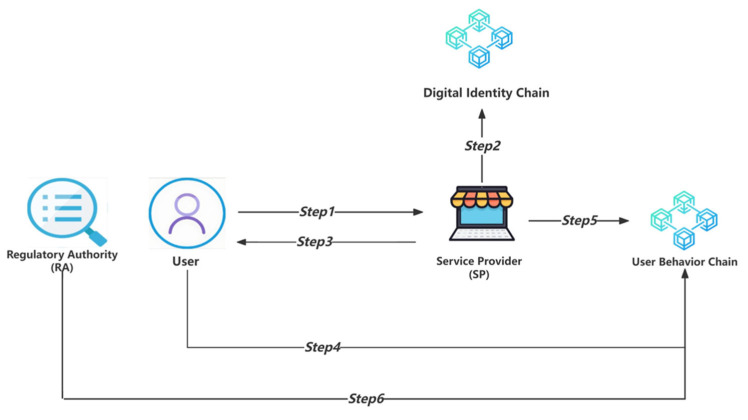
The digital identity usage in the sharing-UID-only scenario.

This scenario is applicable when the service provider only needs to verify the legitimacy of the UID in the user’s digital identity to provide services, meaning such providers do not require additional user personal information when delivering services. The digital identity usage in this scenario will involve six steps, as illustrated in Figure 3.

Step 1: The user sends a login request to the specific service provider (SP), uses the user’s private key (PriU) to digitally sign the current real-time timestamp (Ts1) and current service provider (SPID), obtains PriU (SPID, Ts1) as a zero-knowledge assertion, uses the current service provider’s public key (PubSP) to encrypt their UID and PriU (SPID, Ts1), and sends the obtained PubSP (UID, PriU(SPID, Ts1)) to the service provider. We can describe the process with the following symbolic description:

User → SP: {PubSP(UID, PriU(SPID, Ts1))}(7)

Step 2: The service provider (SP) obtains the PubSP (UID, PriU(SPID, Ts1)) and decrypts it to obtain the UID and PriU (SPID, Ts1); then, it obtains the PubU from the digital identity chain through the smart contract function GetPubKey (UID) based on the UID. The service provider (SP) will use the PubU to verify the PriU (SPID, Ts1) signature. Suppose the timestamp (Ts1) can be successfully obtained within the valid time range, and the SPID matches the SPID of the current service provider. In that case, the verification will be passed, and the user’s identity will be proven. We can describe the process with the following symbolic description:

SP → Digital Identity Chain: {GetPubKey(UID)}(8)

Step 3: If the user’s digital identity is authenticated in Step 2, the service provider (SP) starts to provide services to the user in this step. If authentication fails, the user is required to re-authenticate.

Step 4: The user uploads the authentication success/failure results obtained from the service provider (SP) to the user behavior chain. The relevant data will be encrypted using the public key, PubRA, of the regulatory authority (RA) in the following format:

PubRA(UID, PriU(SPID, Success/Fail, NULL, Ts2))(9)

We can describe the process with the following symbolic description:

User → User Behavior Chain: {PubRA(UID, PriU(SPID, Success/Fail, NULL, Ts2))}(10)

Step 5: The service provider (SP) uploads the success/failure result of the user’s digital identity authentication to the user behavior chain. The relevant data will be encrypted using the public key, PubRA, of the regulatory authority (RA) in the following format:

PubRA(SPID, PriSP(UID, Success/Fail, NULL, Ts3))(11)

We can describe the process with the following symbolic description:

SP → User Behavior Chain: {PubRA(SPID, PriSP(UID, Success/Fail, NULL, Ts3))}(12)

Step 6: The regulatory authority (RA) can access the data on the user behavior chain at any time. Using RA’s private key, PriRA, can review the authentication results of any user for specific service providers and regulate users suspected of suspicious logins or brute force attacks.

#### 3.2.2. Scenario 2: Sharing UID and Specific Personal Identity Category Information

In this scenario, the service provider (SP) aims to deliver effective services by requesting users share specific categories of personal information from their digital identities and authenticating the UID. The personal information verification set {PIV_id, PIV_phone, … PIV_n}, uploaded by users during digital identity registration, plays a crucial role. The system achieves zero-knowledge proof for sharing personal information by utilizing a hash chain verification mechanism.

**Figure 4:**
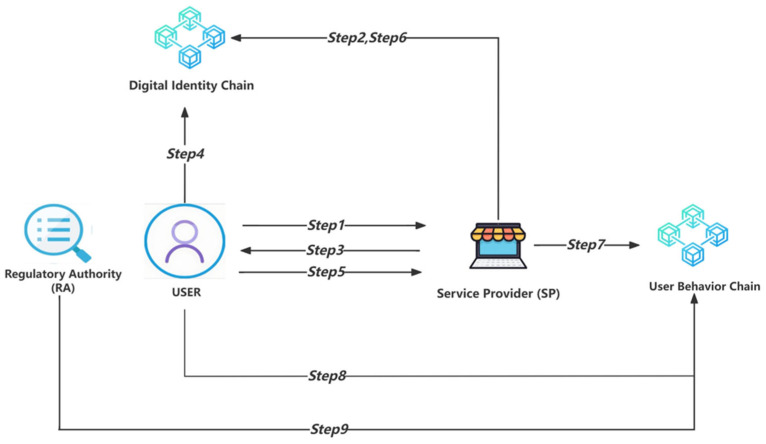
The digital identity usage in sharing UID and specific personal identity category information scenario.

This scenario will be divided into nine steps, as detailed in Figure 4.

Steps 1 and 2 are identical to the corresponding steps in Scenario 1 and will not be reiterated.

Step 3: After confirming the user’s identity, if the service provider (SP) needs to use a certain type of personal information from the user’s digital identity, suppose the service provider requests the user’s identity card information, the following data request is made:

PriSP(PubU(UID, PIN_id, Ts2))(13)

We can describe the process with the following symbolic description:

SP → User: {PriSP(PubU(UID, PIN_id, Ts2))}(14)

Step 4: The user uses the smart contract function GetC(UID, PriU(SPID, PIN_id, Ts3)) to lock the sharing rights of the current personal data category PIN_id to the current service provider through SPID. Determine the remaining verifiable times corresponding to PIN_id. If c > 1, the user can continue providing zero-knowledge proof information to SP; otherwise, the digital identity must be updated first.

We can describe the process with the following symbolic description:

User → Digital Identity Chain: {GetC(UID, PriU(SPID, PIN_id, Ts3))}(15)

In the case of c > 1, the user will calculate and generate Hashx(PI_id·Nonce) locally, where x = c − 1, which means perform c − 1 hashing on PI_id·Nonce. The data will next be used as the assertion for a zero-knowledge proof.

Step 5: The user provides the service provider (SP) with Hashx(PI_id·Nonce). The transmitted data format is as follows:

PubSP(UID, PIN_id, PriU(Hashx(PI_id·Nonce), Ts4))(16)

We can describe the process with the following symbolic description:

User → SP: {PubSP(UID, PIN_id, PriU(Hashx(PI_id·Nonce), Ts4))}(17)

Step 6: The service provider (SP) obtains the returned data PriIC(PriU(Hashc(PI_id·Nonce))) through the smart contract function Fetch(UID, SPID, PIN_id) on the digital identity chain. The service provider (SP) can obtain Hashc (PI_id·Nonce) with the help of the public key PubIC and PubU. We can describe the process with the following symbolic description:

SP → Digital Identity Chain: {Fetch(UID, SPID, PIN_id)}(18)

If the zero-knowledge proof assertion provided by the user in step 5 is true, then the operational relationship between the Hashc(PI_id·Nonce) obtained by the service provider (SP) from the digital identity chain and the Hashx(PI_id·Nonce) provided by the user is as follows:

Hashc(PI_id·Nonce) = Hash(Hashx(PI_id·Nonce))(19)

If the service provider (SP) can obtain the above operation relationship result after performing a hash operation on Hashc(PI_id·Nonce), it proves that the personal information shared by the user is authentic. This is an excellent example of proving your citizenship without revealing your ID information, using zero-knowledge proof technology to support decentralized identity.

Step 7: The service provider (SP) uploads the success/failure result of the user’s digital identity authentication for this session to the user behavior chain. The relevant data will be encrypted using the public key, PubRA, of the regulatory authority (RA) in the following format:

PubRA(SPID, PriSP(UID, Success/Fail, PIN_id, Ts5))(20)

We can describe the process with the following symbolic description:

SP → User Behavior Chain: {PubRA(SPID, PriSP(UID, Success/Fail, PIN_id, Ts5))}(21)

Step 8: The user uploads the authentication success/failure results obtained from the service provider (SP) to the user behavior chain. The relevant data will be encrypted using the public key PubRA of the regulatory authority (RA) in the following format:

PubRA(UID, PriU(SPID, Success/Fail, PIN_id, Ts6))(22)

We can describe the process with the following symbolic description:

User → User Behavior Chain: {PubRA(UID, PriU(SPID, Success/Fail, PIN_id, Ts6))}(23)

Step 9: The regulatory authority (RA) can access the data on the user behavior chain at any time. Using RA’s private key, PriRA, can review the authentication results of any user for specific service providers and regulate users suspected of suspicious logins or brute force attacks.

In the scenario of sharing UID and specific categories of personal information, the solution adopts a unique hash chain design to construct a personal information verification set {PIV_id, PIV_phone, … PIV_n}, ensuring that no plaintext user personal information will be transmitted between the service provider and the user during the sharing process, protecting user privacy, and successfully achieving data sharing between the user and the service provider while keeping the user data “available but invisible”. With the help of the synchronization semaphore design of the GetC() and Fetch() functions, in addition to ensuring that the user authorizes each data sharing, it can also effectively ensure that the hash provided by the user each time is a reliable zero-knowledge proof credential, preventing attackers from replaying the hash due to asynchronous counter c values (such as race conditions caused by multiple service providers simultaneously requesting specific personal data categories of the same user).

**Figure 5:**
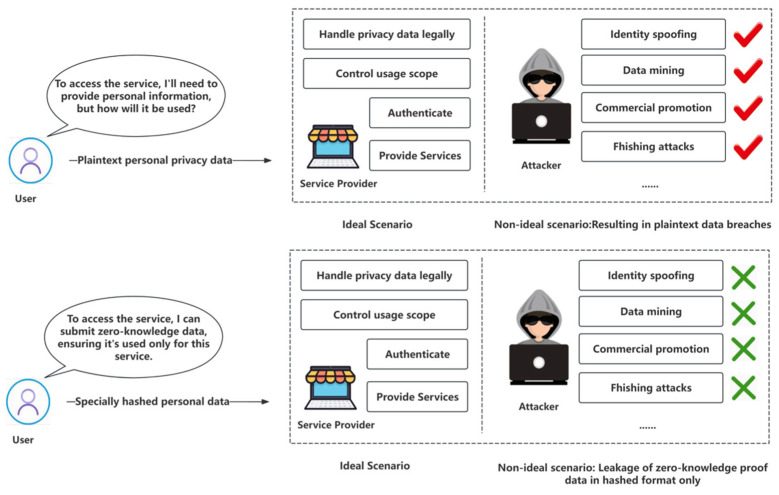
Examples of the advantages of data sharing using zero-knowledge proof technology.

This zero-knowledge proof-based data-sharing technology offers numerous benefits, particularly privacy protection and data misuse prevention. As shown in Figure 5, for example, in our system, a user’s ID information is verified by the IC and converted into a zero-knowledge proof before being uploaded to the digital identity chain. Suppose a service provider requires the user to undergo real-name authentication. When the user provides this zero-knowledge proof to the service provider, the provider can use the information on the digital identity chain to verify the user’s true identity. However, the service provider cannot access the plaintext of this identity, thus preventing misuse of the information, such as for advertising or other commercial purposes. When the IC processes the user’s ID information, it can also create a zero-knowledge proof confirming the “user is of legal age”, which is uploaded to the digital identity chain. If the service provider requests proof of age, the user can provide this zero-knowledge proof. According to the method outlined in our paper, the service provider can verify the user’s legal age via hash validation but cannot obtain the user’s exact age, thus preventing further data mining or tracking. Furthermore, suppose the service provider requires the user to provide their landline number as proof of residency in a specific area. The user can still provide the number through proof of zero knowledge. Based on our description, the service provider can use the information uploaded by the IC to the digital identity chain to validate the authenticity of the zero-knowledge proof, thus confirming the user has a legitimate landline number. However, the provider cannot obtain the actual plaintext of the number, preventing misuse such as harassing or marketing calls.

### 3.3. Stage 3: Digital Identity Update Stage

In our user self-sovereign identity solution, users’ digital identities can be updated. These updates occur in two scenarios: when a service provider (SP) requires the user to share a specific category of personal information that the user has not previously uploaded to their digital identity and when the counter c of PIV_x for a specific personal information category has decreased to 1. We will discuss these two scenarios separately. In this stage, four types of smart contract functions will be utilized, as listed and described in Table 6.

#### 3.3.1. Scenario 1: Service Provider Requests User to Add a Specific Category of Personal Identity Information

When a service provider needs a user to share a specific category of personal information, but the user has not yet added the requested category to their digital identity, this scenario is triggered. In this scenario, the initiating service provider requesting personal information sharing needs to initiate the request through a smart contract function, and other service providers in the same industry vote on the request through smart contract functions. Through this peer review process, it is first determined whether the request should be submitted to the user. Suppose the number of service providers approving the request exceeds a specific threshold. In that case, the request will be forwarded to the user, who will ultimately decide whether to add the category of personal information for sharing. During this process, regulatory agencies will assist in verifying the authenticity of the voting results to ensure the authenticity and effectiveness of the peer review.

**Figure 6:**
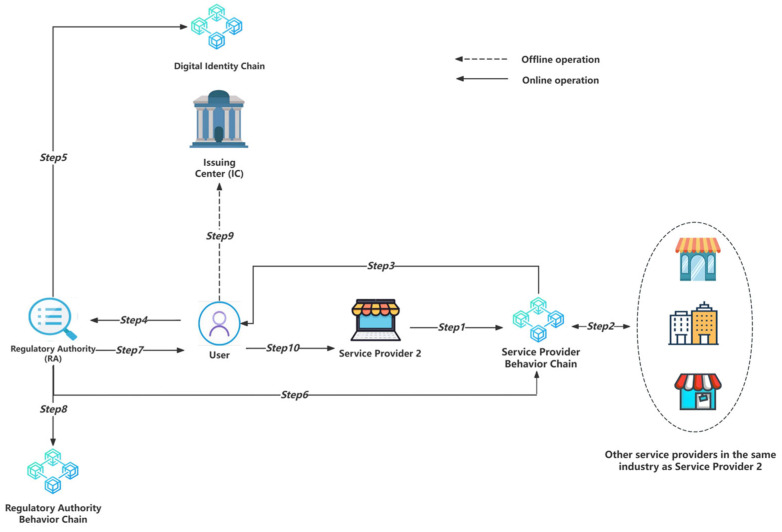
The digital identity update is requesting to add a specific personal identity category information scenario.

This scenario will involve ten steps, as illustrated in Figure 6. We assume that the service provider applying for the addition of the personal information category is SP2, and the service providers participating in this peer review are SP1 to SPn.

Step 1: Service provider SP2 initiates a new personal information sharing request for a specific UID user, along with the request reason parameter Reason and the corresponding personal information name PIN_x. The request is initiated through the following smart contract function:

Request(PriSP2(UID, Reason, PIN_x, Ts1))(24)

We can describe the process with the following symbolic description:

SP2 → Service Provider Behavior Chain: {Request(PriSP2(UID, Reason, PIN_x, Ts1))}(25)

Step 2: The smart contract function initiates a request review to other service providers (SPs) in the industry, and the other SPs determine whether the reason for the request is reasonable in the industry. The SP that approves the request will return its SPID to the service provider behavior chain through the smart contract function in the following format:

Vote(PubSC(PriSPn(SPnID, UID, Ts2)))(26)

We can describe the process with the following symbolic description:

{SP1, SP3, …, SPn} → Service Provider Behavior Chain: {Vote(PubSC(PriSPn(SPnID, UID, Ts2)))}(27)

Step 3: The service provider behavior chain collects the number of SPs and corresponding SPIDs that approve the request through the Vote() function and collects the SPIDs into a LIST. If the number of approvals exceeds a specific set threshold, the application request will be forwarded to the user. The format of the request data to be sent is as follows:

PriSC(PubU(LIST, Reason, PIN_x, SP2ID, Ts3))(28)

We can describe the process with the following symbolic description:

Service Provider Behavior Chain → User: {PriSC(PubU(LIST, Reason, PIN_x, SP2ID, Ts3))}(29)

Step 4: The user has the right to adjudicate the request. If the user agrees to add the personal information, they must notify the regulatory agency (RA) to verify the review results. This process is implemented through data transmission in the following format:

PubRA(PriU(Ts4), UID, LIST, Reason, PIN_x, SP2ID)(30)

We can describe the process with the following symbolic description:

User → RA: {PubRA(PriU(Ts4), UID, LIST, Reason, PIN_x, SP2ID)}(31)

Step 5: The regulatory agency (RA) first obtains PubU from the digital identity chain through the smart contract function GetPubKey(UID) based on the UID and uses PubU to verify the PriU(Ts4) signature. The user’s identity can be proven if the signature passes the verification. We can describe the process with the following symbolic description:

RA → Digital Identity Chain: {GetPubKey(UID)}(32)

Step 6: After authenticating the user’s identity, the regulator RA will make a smart contract function call SamplingVerification(PriRA(PubSC(UID, PIN_x, Reason, LIST, SP2ID, Ts1))) to the service provider behavior chain. This is used to verify the authenticity of the peer review in this application. We can describe the process with the following symbolic description:

RA → Service Provider Behavior Chain: {SamplingVerification(PriRA(PubSC(UID, PIN_x, Reason, LIST, SP2ID, Ts1)))}(33)

The SamplingVerification() function will randomly extract the service provider (SP)ID from the LIST parameter and verify the authenticity of the vote by sending data in the following format to the service provider:

PubSPn(UID, PIN_x, Reason, SP2ID, Ts1)(34)

We can describe the process with the following symbolic description:

Service Provider Behavior Chain → SPn: {PubSPn(UID, PIN_x, Reason, SP2ID, Ts1)}(35)

Step 7: The regulatory agency (RA) will collect the request feedback results from the SamplingVerification() function. If the RA verifies the authenticity of the peer review, the user will be given the Success flag. The specific data format is as follows:

PriRA(PubU(PIN_x, Reason, LIST, SP2ID, Success, Ts5))(36)

We can describe the process with the following symbolic description:

RA → User: {PriRA(PubU(PIN_x, Reason, LIST, SP2ID, Success, Ts5))}(37)

Step 8: The regulatory agency (RA) records the UID, LIST, PIN_x, SP2ID, and Ts4 parameters in this application request to the regulatory authority behavior chain in the following format:

PriRA(UID, LIST, PIN_x, SP2ID, Ts4)(38)

We can describe the process with the following symbolic description:

RA → Regulatory Authority Behavior Chain: {PriRA(UID, LIST, PIN_x, SP2ID, Ts4)}(39)

This means that each ruling process by the regulatory agency will also be auditable by users or service provider entities in the future, effectively preventing regulatory violations.

Step 9: The user goes offline to the Issuing Center IC and repeats steps 4 to 6 in the digital identity registration stage to create a new category of personal identity information.

Step 10: After the new category of personal information is updated, the user will return a Success flag to the SP2 that initiated the request. The specific format is as follows:

PriU(PubSP2(LIST, Reason, PIN_x, SP2ID, Success, Ts6))(40)

We can describe the process with the following symbolic description:

User → SP2: {PriU(PubSP2(LIST, Reason, PIN_x, SP2ID, Success, Ts6))}(41)

SP2 can then use this new category of personal information according to the steps in the digital identity usage stage.

In this scenario, any request from a service provider to add a category for sharing personal information will first undergo peer review by other peer service providers, effectively preventing the risk of service providers overreaching for user information. At the same time, regulatory agencies will actively intervene to verify the authenticity and effectiveness of peer review. Evidence of regulatory actions will be retained in the regulatory agency behavior chain for audit by other entities. Thanks to our robust hash chain setup, no plaintext personal data of users is transmitted or shared in this scenario, maintaining the principle of “usable but invisible” data.

The most significant benefit that this peer review mechanism can offer is the prevention of excessive and inappropriate data requests by a service provider beyond acceptable boundaries. By involving other service providers within the same industry, this mechanism allows for evaluating whether a new category of personal data requested by a specific service provider is necessary. Through this peer review mechanism, all service providers in the industry can benefit. If the data request is deemed reasonable, other service providers can directly request the same data from the user in the future. If the request is deemed unreasonable, the exposure of user data can be reduced through a voting process.

**Figure 7:**
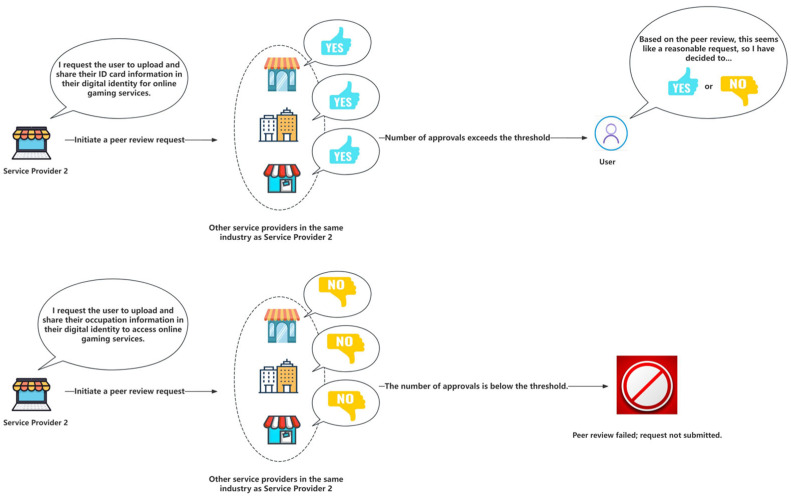
Example of practical application of the peer review mechanism.

As shown in Figure 7, for example, suppose a game service provider is offering services to a new user and, by age restrictions, requires the user to provide age verification. If the user has not provided their ID information in their digital identity, they cannot obtain this proof. Suppose this issue is a first-time occurrence in the industry; in that case, the service provider can initiate a request through the peer review mechanism, asking the user to update their digital identity and provide age information. Suppose other service providers in the industry agree that age verification and data request are reasonable; in that case, they will vote in favor of the request, prompting the user to update their identity and provide the relevant zero-knowledge proof of their age to access the service. Subsequently, other service providers in the gaming industry will not need to initiate another peer review for similar requests and can directly ask the user for the data. This is a reasonable example.

However, after verifying the user’s age meets the game’s requirements, suppose the current game service provider requests the user to update and provide their job and occupation information for the service, which is clearly an unreasonable data request; when this peer review request is made, most of the service providers in the industry will oppose it, and the user will reject the update and provide the data. This mechanism effectively reduces unnecessary data requests.

Additionally, we plan to design a credit-scoring system for each entity in practical applications. This score will start from an initial value, influenced by factors such as past peer review participation, multi-party oversight accountability, and fraudulent activities. Each service provider will have its own credit score, and its credit rating will affect the weight of its opinion in the peer review process. Service providers with a low credit score will be required to stop offering services.

#### 3.3.2. Scenario 2: Specific Category Personal Information Validation Count m Has Decremented to 1

**Figure 8:**
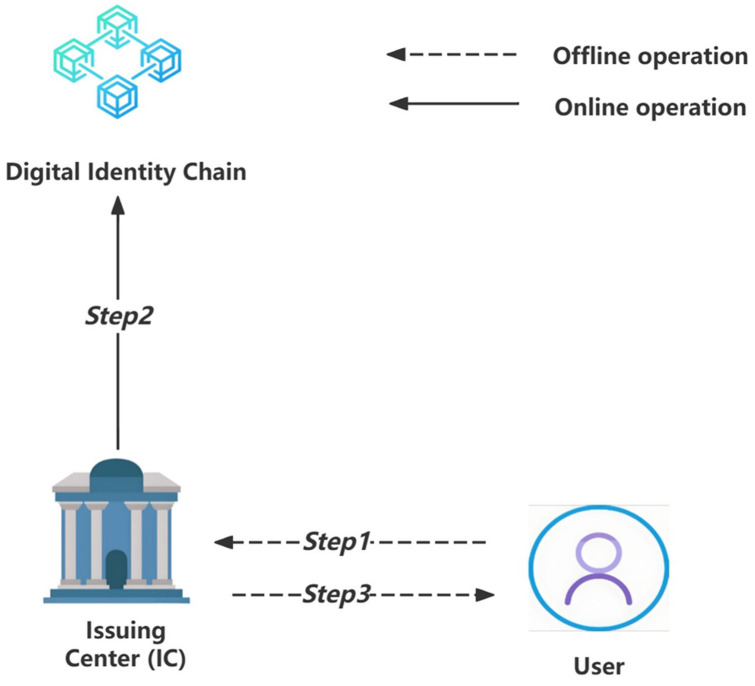
Scenario of updating the validation count for a specific category of personal information by the user in the digital identity update stage.

As mentioned, we utilize a hash chain design to achieve zero-knowledge proof-based sharing of user personal data. In this process, each data-sharing operation requires extracting hash data from the digital identity chain and decrementing the validation count c by 1. When the validation count c is decremented to 1, the hash data extraction operation for this specific category of personal information on the current digital identity chain can no longer provide zero-knowledge proof. Therefore, the user needs to update the personal information of this category in their digital identity. This scenario involves three steps, as shown in Figure 8.

Step 1: The user visits the Issuing Center (IC) offline, provides personal identification, and the personal information to be updated(PI_x). We can describe the process with the following symbolic description:

User → IC: {Physical Identity, PI_x}(42)

Step 2: The Issuing Center (IC) verifies the user’s physical identity, generates a new PIV_x using the new random number parameter Nonce and the PI_x provided by the user, and constructs the following format parameters to call the smart contract function Add() to add the new PIV_x to the digital identity chain.

Add(PriIC(UID, Hash(PubSC(PIV_x), PIN_x, m), Ts), PubSC(PIV_x), PIN_x, m)(43)

We can describe the process with the following symbolic description:

IC → Digital Identity Chain: {Add(PriIC(UID, Hash(PubSC(PIV_x), PIN_x, m), Ts), PubSC(PIV_x), PIN_x, m)}(44)

Step 3: The Issuing Center (IC) provides the user with the new random number Nonce generated in Step 2. The user must securely store this random number, which will be used for subsequent verification steps in the digital identity usage stage. We can describe the process with the following symbolic description:

IC → User: {Nonce}(45)

In this scenario, users can update the zero-knowledge proof verification count for their personal information while ensuring that no plaintext information is transmitted to the digital identity chain throughout the entire process, thereby maintaining the “usable but invisible” nature of user personal information.

### 3.4. Stage 4: Digital Identity Deletion Stage

In self-sovereign identity, users have complete control over their digital identities. Therefore, users can choose to terminate the sharing of certain personal information at any time and swiftly delete related data through a smart contract function. This stage will involve only one smart contract function, as detailed in Table 7.

**Figure 9:**
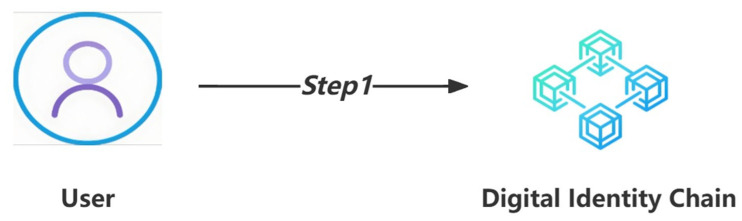
Digital identity deletion stage.

This stage will consist of only one step, as illustrated in Figure 9.

Step 1: As the data sovereign party, the user actively terminates the sharing of certain digital identity information by constructing the following parameters and calling the smart contract function Delete():

Delete(UID, PriU(PIN_x, Ts))(46)

We can describe the process with the following symbolic description:

User → Digital Identity Chain: {Delete(UID, PriU(PIN_x, Ts))}(47)

However, as we all know, data on the blockchain cannot be deleted. Our research recommends using the Chameleon hash algorithm to modify the on-chain counter c value to 0 [36,37,38,39,42,43,44], making the relevant digital identity information unusable. Utilizing the Chameleon hash algorithm, the data remain tamper-proof on the blockchain while allowing the change in specific hash values to ensure that the original information cannot be restored. On the other hand, since the user’s on-chain data are encrypted and protected by PubSC. In this way, as long as the counter c value is set to zero, even if malicious actors illegally access the encrypted data on the blockchain, they cannot obtain any useful information.

Our research focuses on blockchain-based self-sovereign identity generation technology, highlighting several innovative approaches:

Innovation 1: Zero-knowledge proof for privacy protection

We propose a zero-knowledge proof (ZKP) technology to address privacy concerns in information sharing under the self-sovereign identity framework. ZKP allows verifiers to confirm the truth of a statement without revealing specific details, ensuring perpetual privacy during multi-party computation [45,46,47,48,49]. This approach makes information elements “usable but not visible”, filling critical gaps in privacy protection. Although various attack techniques against hash algorithms exist, the irreversibility of hash algorithms remains trustworthy, provided secure hash functions are chosen [50,51,52,53,54].

Innovation 2: Blockchain-based mutual supervision mechanism

We introduce a three-party mutual supervision mechanism to oversee interactions in the autonomous identity generation process. Regulatory agencies can effectively monitor behaviors on the blockchain, ensuring transparency and accountability among all parties involved in information sharing. This mechanism curbs dishonest behaviors and reinforces regulatory oversight, enhancing trust in identity transactions.

Innovation 3: Peer Review Mechanism for Data Request Validation

We propose a peer review mechanism to mitigate risks associated with excessive data requests. Before requesting new categories of personal information, service providers undergo industry peer review to justify data necessity. Automated through smart contracts, this process includes verification by regulatory authorities to maintain integrity and prevent misuse.

Compared to existing SSI systems, our solution offers unique advantages in privacy protection, preventing data misuse, multi-party behavioral privacy oversight, and reducing excessive data requests based on innovations. Specifically, unlike Sovrin and ShoCard, our zero-knowledge data-sharing approach eliminates the possibility of users sharing plaintext data. This ensures privacy protection when interacting with current service providers. Additionally, since the shared zero-knowledge data does not contain any user plaintext information, the service provider is prevented from misusing data beyond the scope of this interaction. This addresses the persistent data security issue of service providers “owning the data once and using it repeatedly”, thus strengthening the user’s control over their self-sovereign identity. Moreover, our introduced multi-party supervision mechanism ensures that all actions by entities are accurately recorded and audited, significantly reducing the potential for malicious activities by any entity. These descriptions are equally applicable when comparing uPort, Veramo, TCID, DT-SSIM, PT-SSIM, and SISSI.

INCHAIN and AASSI, on the other hand, enhance the oversight mechanisms, involving one-way regulation from one type of entity to another, such as insurance companies overseeing claim initiators (INCHAIN) or trackers and issuers overseeing users (AASSI). However, one-way regulation cannot prevent global malicious behavior, as the actions of unregulated entities may remain uncontrolled. Our multi-party mutual oversight mechanism addresses this risk. Furthermore, the issues of privacy protection, data misuse prevention, and excessive data requests still persist in INCHAIN and AASSI, making the comparisons mentioned earlier still applicable.

**Figure 10:**
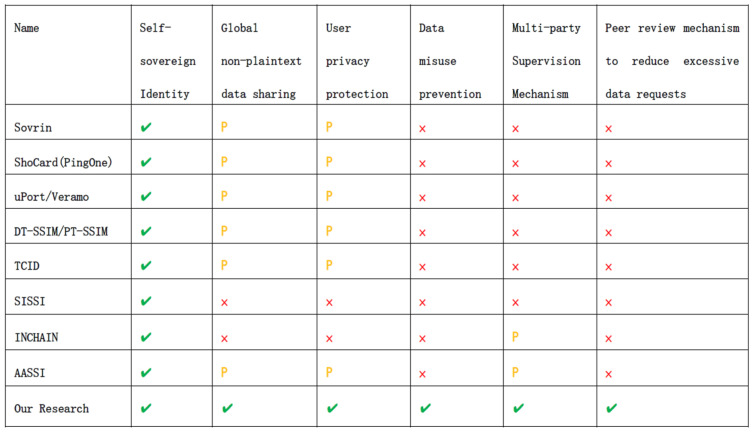
Comparison of innovations with existing work. √ represents full compliance, × represents non-compliance, and P represents partial compliance.

We have presented the relevant projects in image format to facilitate the comparison. As shown in Figure 10, our innovations and advantages are compared with existing solutions.

## 4. Experiments and Analysis

### 4.1. Formal Security Verification of Protocols Based on Scyther

Scyther 1.1.3 is an automated security protocol verification tool released by the CISPA Helmholtz Center for Information Security in Germany [55,56,57]. It is used to identify potential attacks and vulnerabilities in protocols and has been widely used by researchers worldwide to verify various security protocols.

**Figure 11:**
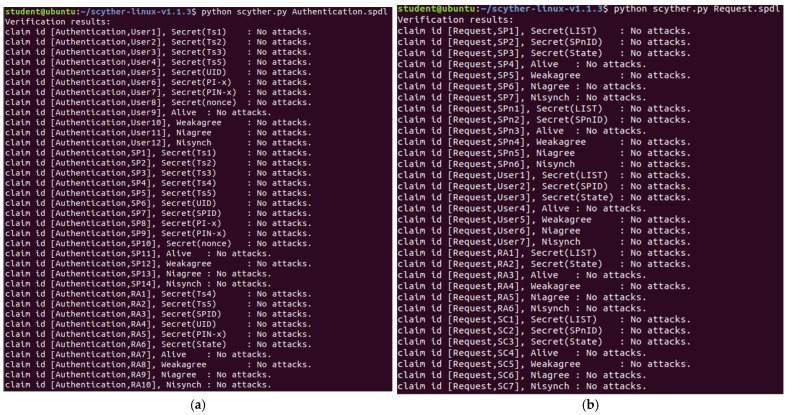
(**a**) Protocol security verification results for digital identity usage stage, analyzed using Scyther; (**b**) protocol security verification results for digital identity update stage, analyzed using Scyther.

Figure 11 presents our research’s formal verification results for the digital identity usage stage (Figure 11a) and digital identity update stage (Figure 11b), which were analyzed using Scyther.

The formal verification methodology of Scyther evaluates the following security properties in our proposed solution:

✓ Confidentiality
✓ Aliveness
✓ Weak Agreement
✓ Non-injective Agreement (Niagree)
✓ Non-synchronization (Nisynch)
✓ State Consistency

Confidentiality effectively prevents information leakage, meaning attackers cannot access sensitive data transmitted within the protocol (such as passwords, keys, session tokens, etc.), thereby protecting user privacy and system security. Aliveness and Weak Agreement ensure that the communicating parties are genuine, trusted entities, preventing impersonation by a man-in-the-middle (MITM) attacker. These properties guarantee that both parties can confirm their participation in the protocol and avoid “communicating” with non-existent entities. Non-injective Agreement (Niagree) ensures that attackers cannot reuse previously valid messages to deceive protocol participants, meaning each communication event is unique and fresh, preventing replay attacks where old transaction data are reused. Non-synchronization (Nisynch) ensures that attackers cannot disrupt protocol logic by altering the message order, such as forging and sending response messages without an actual request. This property protects the multi-step interaction process within the protocol, ensuring that execution steps are not tampered with. Finally, State Consistency ensures that state variables in the protocol (such as session identifiers and key states) are not tampered with or compromised by attackers, preventing session hijacking and impersonation of legitimate users.

Currently, the preliminary planned communication methods have not shown any detected known risks.

### 4.2. The Analysis of Correctness and Interpretation of Experimental Results

We will address concerns and apprehensions from the perspectives of users, service providers, and regulatory bodies, respectively, and demonstrate, based on the experimental results and research design, that relevant issues will not arise. This ensures that the research design meets these security benchmarks.

#### 4.2.1. Concerns from the Perspective of Honest Users

From the user’s perspective, they often have the following concerns:

1. Identity Theft or Substitution: Users worry that attackers might steal or replace their authentication credentials, leading to unauthorized access or impersonation.
2. Data Leakage During Transmission: Users are concerned that personal data transmitted over the network could be intercepted or disclosed, leading to privacy breaches.
3. Infringement of Data Sovereignty: Users fear losing control over their data through unauthorized access or service providers exerting undue control without consent, such as using personal data on the blockchain without authorization or attempting to use data categories declared as deleted.
4. Engagement in Malicious Activities: Users worry about unintentionally engaging in malicious activities while using the service, potentially causing harm to others or the system.
5. Denial of Malicious Behavior: There is concern that dishonest users may deny engaging in malicious activities, making it difficult to hold them accountable for misconduct or security breaches.

#### 4.2.2. Security Analysis of the System for the Users’ Concerns

1. Identity Theft or Substitution.

If an attacker aims to steal or impersonate an identity, they typically have two common approaches: forging identity credentials or hijacking an existing legitimate user’s identity. If the attacker chooses the first option, they will need to construct the necessary forged data to bypass the identity authentication step labeled as Equation (7) in Section 3.2.1.

In this authentication step, the legitimate user provides their UID as a digital identity credential and includes a digital signature containing a timestamp and the target service provider’s ID as a method for validating their digital identity. All the data are encrypted using the service provider’s public key. In this process, if an attacker, named C, aims to forge an identity, C must first perform a man-in-the-middle attack to deceive the user into using the C’s public key for encryption. Only then would the attacker have the opportunity to obtain PriU(SPID, Ts1) and replace the UID in a fake message constructed by C. After replacing the UID, the attacker can either attempt a replay attack using the legitimate digital signature in the packet or forge a new digital signature for the attack. We will reason through both methods, assuming the attacker has successfully carried out the man-in-the-middle attack.

If the attacker opts to replace the UID with a different one, call it UIDX, in a fake message, such as the following:

PubSP(UIDX, PriU(SPID, Ts1))(48)

The UIDX will be used by the service provider to retrieve the user public key from the digital identity chain. But UIDX will not allow the service provider to obtain the correct public key for the user, causing the service provider to fail to verify the digital signature in the data packet, resulting in an authentication failure.

If the attacker chooses to replace the UID and forge the digital signature, i.e., in a message such as the following:

PubSP(UIDX, PriY(SPID, Ts1))(49)

If the chosen UIDX is valid in the digital identity chain, the private key, PriY, used here should be the related to UIDX, namely PriX, in order to construct a valid digital signature. This is clearly very difficult. If the attacker chooses to forge an invalid UIDX, since the UIDX has not been registered with the IC, the service provider will be unable to retrieve the corresponding public key from the digital identity chain, making it impossible to verify the digital signature.

Therefore, it would be extremely difficult for the attacker to successfully forge the user’s identity. Furthermore, the Scyther analysis results also strongly support the conclusion that the difficulty of carrying out a man-in-the-middle attack is high, ensuring that our solution effectively prevents identity theft or impersonation.

2. Data Leakage During Transmission.

For users, the greatest concern during data transmission is the potential leakage of PI_x-related information. Although PI_x is never transmitted or used in plaintext during actual transmission and operation, it can still raise concerns. The online interaction involving PI_x is outlined in the authentication step marked as Equation (17) in Section 3.2.2.

We use PIN_id as an example to explore its logical security. In this process, the PIN_id is hashed together with a Nonce multiple times, and the resulting data are then digitally signed by the user along with the current timestamp. Finally, it is encrypted using the service provider’s public key. It is important to note that this data transmission process has a logical context, as it represents the third interaction step described in Section 3.2.2. To perform this interaction, both the user and the service provider must first complete the previous two steps.

In this context, if an attacker aims to obtain the hash data related to PIN_id, they must first compromise or hijack the current session. If the attacker chooses to break the encryption, they will need to decrypt the information encrypted with the service provider’s public key without having access to the service provider’s private key, which is practically unfeasible within a reasonable timeframe with current technology. Alternatively, if the attacker opts to hijack the session, they must first establish a valid man-in-the-middle attack channel and complete the previous two interactions to acquire the necessary context for obtaining the data. This requires successfully breaking the logic in our previously mentioned Equation (7), which we have already reasoned to be extremely difficult to accomplish. Therefore, it can be logically proven that the difficulty of user data leakage is high, and the analysis results from Scyther further substantiate our argument.

3. Infringement of Data Sovereignty.

In our research design, service providers obtain hashed personal data (PI_x), protected by the public key of a smart contract on the digital identity chain. Security analysis using asymmetric algorithms demonstrates that unauthorized access by service providers is impossible. Even if a service provider attempts to access personal data categories declared as deleted by users, these data, though still existing on the blockchain, remain inaccessible due to the public key of smart contract protection. This ensures users’ self-sovereignty over their data, meeting the security benchmarks of the research.

4. Engagement in Malicious Activities.

When the user interacts with the service provider, the relevant user data are submitted to the User Behavior Chain by both the user and the service provider. If the data from both parties is inconsistent, it will trigger an intervention by the regulatory authority. In the case where a dishonest user or service provider attempts to engage in malicious activities, they would need to find ways to tamper with the data submitted by the other party to the User Behavior Chain. However, due to the immutability of blockchain, they cannot alter the data that has already been recorded on the chain. Therefore, their only option would be to attempt to intercept and modify the process by which the data are submitted to the chain.

The interaction logic for submitting data to the blockchain is as follows:

User → User Behavior Chain: {PubRA(UID, PriU(SPID, Success/Fail, NULL, Ts2))} SP → User Behavior Chain: {PubRA(SPID, PriSP(UID, Success/Fail, NULL, Ts3))}(50)

In this process, dishonest users or service providers would need to forge the other party’s digital signature in order to successfully tamper with the data. Clearly, a forged digital signature cannot pass decryption and validation by the regulatory authority using the legitimate public key, which would trigger regulatory review. Furthermore, attempting to forge a valid digital signature without access to the other party’s private key is virtually impossible with current technology. This logically demonstrates that, in our proposed solution, it is extremely difficult for either users or service providers to engage in unilateral malicious behavior.

5. Denial of Malicious Behavior.

As mentioned earlier, when users utilize the services provided by service providers, their relevant user data are submitted by the service provider to the user identity regulatory chain. Considering the immutable nature of blockchain, users cannot erase or tamper with pertinent information. This implies that users have undeniable accountability for their actions, meeting the security benchmarks of this study.

#### 4.2.3. Concerns from the Perspective of Service Providers

From the perspective of service providers, they often have the following concerns:

1. Identity theft or substitution: service providers are worried about unauthorized access to user accounts due to identity theft or substitution, leading to security breaches and misuse of user data.
2. Fabricated personal data: there is concern that users may submit false personal information, compromising data accuracy and service quality.
3. Excessive data collection: service providers are cautious about over-collecting user data, potentially infringing privacy rights and violating regulations.
4. Abuse of user data: service providers fear misuse of collected data, such as unauthorized sales to third parties or usage for purposes unrelated to the service, leading to privacy violations and loss of trust.
5. Identity fraud: there is a risk of malicious actors impersonating legitimate service providers to access user data or deceive users into providing sensitive information.
6. Denial of malicious behavior: service providers may deny involvement in malicious activities or responsibility for data breaches, undermining trust and accountability.

#### 4.2.4. Security Analysis of the System for the Providers’ Concerns

Based on the experimental results and research design description, we can demonstrate that the concerns will not materialize. This can be substantiated through the following analysis:

1. Identity Theft or Substitution.

When discussing user concerns, we analyzed the risk of identity theft or substitution. By leveraging timestamps, asymmetric encryption algorithms, and Scyther’s secure evaluation of all UID-related claims, we can demonstrate that our research scheme mitigates user authentication identity theft or substitution issues. This argument also holds from the service providers’ perspective, meeting the study’s security benchmarks.

2. Fabricated Personal Data.

In our research design, since users’ UID and all personal information are verified offline and stored on the blockchain by the authentication center (IC), and all data on the blockchain include a digital signature from the IC, it implies that user data on the blockchain is trustworthy. Providing fabricated information is highly unlikely unless users can forge the IC’s digital signature. Forging a digital signature in an asymmetric cryptographic system is theoretically impossible without a legitimate private key.

3. Excessive Data Collection.

Due to the presence of a peer review mechanism, if a service provider wishes to further access specific personal information of a user, and this category of information has not been requested by other service providers, the request must first undergo peer evaluation and voting among industry peers. This will effectively prevent service providers from excessively soliciting unnecessary user information, thus meeting the security benchmarks of the study.

4. Abuse of User Data.

Past data misuse often involves service providers obtaining users’ personal information in plaintext and applying the relevant data outside the scope of authorization without obtaining user consent. In our research, however, since all user personal information is hashed data with random nonce values, the data can only be used for the intended purpose. They cannot be utilized for any other purposes. Additionally, the irreversible nature of the hash function prevents service providers from forging hashes for other purposes. Using the formula below, we can demonstrate the infeasibility of deriving the m-th hash from the (m + 1)-th hash.

When considering the derivation from the (m + 1)-th hash to the m-th hash, let us assume we have a hash function H, which maps the input x_m+1_ and random number Nonce_m+1_ to the hash value h_m+1_:

h_m+1_ = H(x_m+1_, Nonce_m+1_) (51)

Now, we want to derive the m-th hash h_m_ from the (m + 1)-th hash h_m+1_. Due to the irreversibility of the hash function, we cannot directly reverse-engineer the original inputs x_m_ and Nonce_m_, meaning we cannot find H^−1^ (h_m_, Nonce_m_).

Therefore, the infeasibility of deriving the m-th hash from the (m + 1)-th hash is determined by the one-way nature of the hash function, making it impossible to reverse-engineer through any formula. This also implies that service providers cannot misuse user data, thus meeting the security benchmarks of this study.

5. Identity Fraud.

According to our design, if an attacker intends to impersonate a service provider to steal user data, they will need to convince the user that they possess a legitimate service provider identity during the following interactions:

SP → User: {PriSP(PubU(UID, PIN_id, Ts2))} SP → User Behavior Chain: {PubRA(SPID, PriSP(UID, Success/Fail, PIN_id, Ts5))}(52)

In any of these interaction processes, the attacker would need to successfully forge a digital signature that can be validated by the user or the regulatory authority. However, forging a legitimate digital signature without the other party’s private key is difficult to achieve with current technology, thus logically proving that the service provider’s identity in our solution is hard to counterfeit.

6. Denial of Malicious Behavior.

Like the denial of malicious behavior from the user’s perspective, since service providers’ requests for user data are recorded on the service provider behavior chain, considering the immutable nature of the blockchain, service providers are also unable to deny their actions, ensuring non-repudiation. Thus, this meets the study’s security benchmarks.

#### 4.2.5. Concerns from the Regulatory Authority’s Perspective

From the regulatory authority’s perspective, the concerns are as follows:

1. Inability to monitor malicious behavior: regulatory authorities may struggle to effectively monitor and detect malicious activities from both users and service providers, including fraudulent and deceptive practices.
2. Peer review mechanism failures: concerns arise when service providers collude to manipulate the peer review process, rendering it ineffective in identifying and penalizing unethical behavior.
3. Fabrication of peer review results: service providers falsifying peer review outcomes can undermine the credibility and reliability of the peer review process, affecting trust in industry self-regulation.
4. Sensitive data leaks: Regulatory authorities must ensure the confidentiality and security of sensitive information exchanged during communications with users and service providers. Breaches could lead to identity theft, financial fraud, or reputational damage.
5. Tampering with regulatory data: unauthorized alteration or manipulation of regulatory data can undermine its accuracy and reliability, leading to incorrect regulatory decisions or enforcement actions.
6. Leakage of regulatory data: If regulatory data contains sensitive information, there is a risk of compromising user or service provider privacy, potentially harming their rights and interests.
7. Internal malicious behavior: Regulatory authorities must guard against internal misconduct or corruption, as failure to detect such behavior can erode public trust and undermine the authority’s effectiveness.

#### 4.2.6. Security Analysis of the System for the Regulator’s Concerns

Based on the experimental results and research design description, we can demonstrate that the concerns will not materialize. This can be substantiated through the following analysis:

1. Inability to Monitor Malicious Behavior.

All actions of users and service providers are comprehensively recorded on the blockchain, providing regulatory authorities with a global perspective to exercise their responsibilities, thus eliminating regulatory blind spots and meeting the study’s security benchmarks.

2. Peer Review Mechanism Failures.

Regulatory authorities review voting results to prevent collusion attacks. They investigate and confirm that service providers genuinely participated in evaluations independently, ensuring immediate exposure of collusion and meeting the study’s security benchmarks.

3. Fabrication of Peer Review Results.

Regulatory oversight and the inclusion of digital signatures in each vote prevent the falsification of peer review results. This ensures the integrity of the process and aligns with the study’s security benchmarks.

4. Sensitive Data Leaks.

Scyther experimental results indicate secure data exchanges between regulatory authorities and users or service providers. Asymmetric encryption ensures that attackers cannot decrypt related ciphertexts, preventing sensitive information leakage and meeting the study’s security benchmarks.

5. Tampering with Regulatory Data.

All regulatory data are sourced from the blockchain, ensuring data integrity due to the blockchain’s immutability. This meets the study’s security benchmarks.

6. Leakage of Regulatory Data.

Interactions between regulatory authorities, users, and service providers are secure. Regulatory data on the blockchain are encrypted using the regulatory authority’s public key, ensuring that only regulatory authorities can access plaintext data. This prevents data leakage and meets the security benchmarks.

7. Internal Malicious Behavior.

Regulatory actions are recorded on the blockchain, ensuring non-repudiation. Users and service providers can audit regulatory actions, preventing regulatory authorities from concealing malicious behavior. This meets this study’s security benchmarks.

### 4.3. Simulation Analysis of Computational Efficiency

We identified the steps involving asymmetric cryptography for encryption and digital signing to assess the efficiency of the cryptographic algorithms in the research design. We recorded the types and frequencies of cryptographic operations used in each step. The statistical results are presented in Table 8.

Based on these data, we measured the execution time of all encryption and digital signature operations in the online process through simulation tests. These simulations were conducted using test code written in Python 3.9.2 (interpreted language) and C (compiled language), both based on the RSA asymmetric encryption algorithm. The tests involved cryptographic calculations such as encryption and digital signing, with the test data size based on the expected data volume for each step. The tests were conducted under the following conditions, with each test executed 10 times and the average value taken.

- Operating System: Debian 5.5.17-1kali1 (21 April 2020) x86_64 GNU/Linux
- CPU: AMD Ryzen R5 5600H (configured to four cores and four threads via VMware to simulate limited resources) (Advanced Micro Devices (AMD) based in Santa Clara, CA, USA)
- Memory: DDR4 4 GB 2666

**Figure 12:**
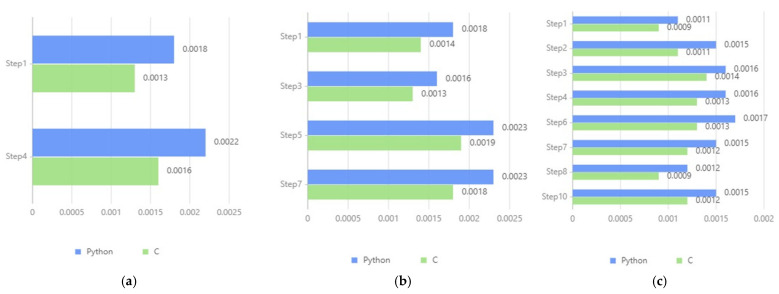
(**a**) Steps with cryptographic operations and their execution time in scenario 1 of the digital identity usage stage; (**b**) steps with cryptographic operations and their execution time in scenario 2 of the digital identity usage stage; (**c**) steps with cryptographic operations and their execution time in scenario 1 of the digital identity update stage. All time units in this Figure are in seconds.

The efficiency test results for the digital identity usage and update stages are shown in Figure 12. While computation times vary due to differences in parameters and data volumes for each step, the overall results demonstrate that even with limited hardware, cryptographic operations in both interpreted and compiled languages maintain acceptable millisecond-level speeds, with compiled languages offering better performance. In real-world scenarios with better hardware, the computational efficiency of this design is expected to meet everyday application requirements.

## 5. Conclusions

This paper briefly introduces research on blockchain-based peer-supervised self-sovereign identity generation and privacy protection technologies. This study proposes a blockchain-based self-sovereign identity mechanism, innovatively achieving “available but invisible” user digital identity personal data. A multi-party mutual supervision mechanism ensures the security of interactions among data-sharing entities and implements effective peer review controls to address risks of potential data over-retrieval and misuse by service providers.

In future work, we plan to refine the following details of this solution:

Precision of biometric matching: Since personal information is stored on-chain in hashed form, the data comparison is also in the form of hashed data, which is suitable for textual data. However, the hashed comparison may fail for biometric data such as fingerprints and irises, which have a range of accuracy. Our theoretical requirement is that the biometric collection method used during digital identity registration matches the one used during digital identity usage with a fixed accuracy rate. However, this theoretical consideration may face limitations, and this will be a focus area for improvement in the future.

Enhancing SSI interoperability and expanding use cases: Interoperating with other SSI systems remains challenging due to differences in Decentralized Identifier (DID) formats across various blockchains, algorithms, and application scenarios. To address this, we are designing a scalable solution where digital identity authentication and sharing resemble MPLS in computer networks. By adding labels to data packets, different SSI systems can use unique labels, allowing us to process and parse data accordingly for improved interoperability. Additionally, we plan to expand SSI use cases beyond design to practical applications, such as privacy protection against tracking and inference attacks, and offline scenarios like using SSI identities for receiving packages without revealing a user’s physical identity.

Finally, advanced proof of system security features using formal or mathematical methods, automatically or semiautomatically, is also a challenging research realm for designed systems. In Section 4, we sketched some security features that are intuitively justified but challenging to prove in a customized way using proper tools and mechanisms, like a theorem prover [58]. These are our future works.

## 6. Patents

Patent No. ZL 2023 1 1600886.9 (CN) has resulted from the research presented in this study.

## Figures and Tables

**Table 1:** Names and descriptions of the entities.

Entity Name	Description
User	Users are physical entities who register and use digital identities.
Issuing Center (IC)	The Issuing Center (IC) is an entity in the digital identity registration stage that is the only entity capable of creating and updating digital identity information. In our research, the Issuing Center (IC) acts as a trusted entity, like a government institution, responsible for verifying users’ physical identities and creating their digital identities. The IC’s digital signature endorses the identity information created. However, the IC does not control any blockchain or the information on the blockchain.
Service Provider (SP)	The Service Provider (SP) is an entity that interacts with users for digital identity transactions. It verifies the user’s digital identity and provides specific services to the user, during which it requires user data.
Regulatory Authority (RA)	The Regulatory Authority (RA) regulates digital identity registration and usage, ensuring compliance and security of the digital identity system.

**Table 2:** Names and descriptions of the blockchains.

Blockchain Name	Description
Digital Identity Chain	The Digital Identity Chain records user digital identity information. In the digital identity registration stage, the user’s digital identity information is stored on the Digital Identity Chain.
User Behavior Chain	The User Behavior Chain records user behavior during the digital identity usage stage.
Regulatory Authority Behavior Chain	The Regulatory Authority Behavior Chain records the actions of the Regulatory Authority (RA) during digital identity registration and usage processes.
Service Provider Behavior Chain	The Service Provider Behavior Chain records SP behavior during the digital identity usage and update stage.

**Table 3:** Names and descriptions of the symbols used in Section 3.

Symbol	Description
PubX	The public key of a particular entity. For example, PubU represents the user’s public key.
PriX	The private key of a particular entity. For example, PubU represents the user’s private key.
UID	Stands for User Digital Identity ID. An IC issues it and is unique.
SPID	Stands for Service Provider ID. It is assigned by an IC when the SP is established and is unique.
PI_x	PI_x represents the detailed content of a specific type of user’s personal information. For example, PI_id can represent the user’s identity ID details, and PI_phone can represent the user’s phone number information.
PIN_x	PIN_x denotes the name of a specific type of personal information, such as ’Identity ID’ for PIN_id and ’Phone Number’ for PIN_phone. It categorizes on-chain stored personal information, which remains non-plaintext in this study. Service providers refer to PIN_x when requesting access.
PIV_x	PIV_x represents Personal Information Verification data, which is the hashed personal information ultimately stored on the blockchain. This ensures privacy while supporting zero-knowledge proof sharing and verification. For example, PIV_id represents the verifiable identity ID data. Each PIV_x is a specially processed data set that allows for m instances of zero-knowledge proof verification.
m	The number of hashes used to create PIV_x represents the number of zero-knowledge proof verifications available on the blockchain.
c	c is the counter used by the smart contract’s Fetch() function, which sets up a separate counter for each type of PIV_x for every user, with each counter initialized to m. The value of c can be stored on-chain and modified using a chameleon hash [36,37,38,39] or managed as an internal counter within the smart contract’s storage.
Hashx	The hash operation, where x represents the number of times the hash is applied.
Nonce	The random value hashed with PI_x to create PIV_x is given to the user for future zero-knowledge proof verifications and to prevent replay attacks on the blockchain.
Ts	Timestamp
Success/Fail	Flag for successful or failed operation
Reason	The rationale submitted by the service provider in the peer review mechanism for requesting data serves as the basis for the peer review process.
LIST	The collection of Service Provider IDs in the peer review process who approve the current data request.

**Table 4:** Names and descriptions of the smart contract functions used in the digital identity registration stage.

Smart Contract Function Name	Description
Create () Input: Accepts one parameter pair consisting of the UID and PubU, both signed using the Issuing Center’s (IC) private key. Output: Return True on success, False on failure.	Create a new user digital identity block and write UID and PriIC (UID, PubU) to the block.
Add() Input: Accepts four parameters: the UID and timestamp (Ts), along with the combined hash result for PubSC (PIV_x), PIN_x, and m, all signed with the Issuing Center’s (IC) private key; the PIV_x set is encrypted with the public key of the smart contract function; the digital identity name (PIN_x); and the number of verifiable attempts (m)”. Output: Return True on success, False on failure.	Upload the newly created PIV_x to the digital identity chain.

**Table 5:** Names and descriptions of the smart contract functions used in the digital identity usage stage.

Smart Contract Function Name	Description
GetPubKey() Input: Accepts one parameter, which is UID. Output: Return PubU on success, Null on failure	Find the corresponding PubU on the chain based on the UID and return it as the return value.
Fetch() Input: Accepts three parameters, namely UID, SPID, and personal information name PIN_x. Output: If the input SPID is the same as the currently locked SPID of the GetC() function, the value of counter c is determined. If the counter c is greater than 1, PriIC(PriU(Hashc(PI_x·Nonce))) is returned. A null value is returned if c does not exist; if c equals 1, a data update prompt string is returned. Service is denied if the input SPID is different from the currently locked SPID of the GetC() function or is not locked.	This function and the GetC() function build a synchronization semaphore with each other. If the GetC() function is not called by the user in advance and locks the specific SPID, the current function will not work. To ensure the consistency of the c value each time data are shared, the user should authorize this sharing. When the SPID is acknowledged, this function uses the UID and PIN_x to locate the block containing the corresponding PIV_x data. The smart contract chain code includes counter c, private key PriSC, and corresponding decryptor. Counter c is initially set to the current m value of PIV_x. Each time the smart contract decrypts PubSC(PriIC(PriU(Hashc(PI_x·Nonce)))) and returns PriIC(PriU(Hashc(PI_x·Nonce))), the counter c decreases by 1. When the counter c decreases to 1 for the current PIV_x, it indicates that all the hash data of the current PIV_x have been used and need to be updated. The value of c can be stored on-chain and modified through decrementing using a chameleon hash [36,37,38,39], or it can be managed as an internal counter within the smart contract’s own storage.
GetC() Input: Accepts two parameters, which are the UID and the SPID, PIN_x, Ts combination signed using the private key PriU corresponding to the UID. Output: Returns the current remaining number of verifiable times (c) for the user’s digital identity PIN_x. At the same time, the current c value is locked, and other service providers other than the input SPID are prohibited from initiating data-sharing requests during this period until the Fetch() function confirms that the current sharing has been completed. Negative constant if PIN_x has not been registered.	GetC() confirms the current remaining number of verification times c of the user’s digital identity PIN_x based on the UID and reserves the current number of verification times c for the SPID in the user’s signature. During this period, only the current SPID can initiate a sharing request for PIN_x until Fetch() function is called and confirms that the sharing is completed.

**Table 6:** Names and descriptions of the smart contract functions used in digital identity update stage.

Smart Contract Function Name	Description
Add()	As introduced in Table 4 of the digital identity registration stage
Request() Input: Accepts one parameter in the format PriSP(UID, Reason, PIN_x, Ts). Output: Returns True on success, False on failure.	When a service provider needs to request a user to add a new category of personal information, the Request() function is initiated on the service provider behavior chain using the specified input format.
Vote() Input: Accepts one parameter in the format PriSPn(PubSP(SPnID, UID, Ts)). Output: Returns True on success, False on failure.	When the smart contract function Request() is called, the Vote() smart contract function will automatically initiate a peer review process involving other peer service providers (SP1 to SPn) of the service provider that called the Request() function. For those service providers that approve the application, the Vote() function will be called using the specified input format to cast their votes.
SamplingVerification() Input: Accepts 1 parameter in the format PriSRA(PubSC(UID, PIN_x, Reason, LIST, SPID, Ts)) Output: A collection of verification results returned by the service provider being randomly checked.	This function is used by the regulatory agency (RA) to conduct random checks on the service providers who voted in favor of the application to verify whether they actually participated in the vote and approved the application. This function will randomly select some service providers from the LIST that approve the application and ask these service providers to confirm their actual participation in this vote by sending data in the following format: PubSPn(UID, PIN_x, Reason, SPID, Ts)

**Table 7:** Names and descriptions of the smart contract functions used in digital identity deletion stage.

Smart Contract Function Name	Description
Delete() Input: Accepts two parameters: the UID and the set of personal information name PIN_x and timestamp signed by the corresponding user’s private key. Output: Returns True on success and False on failure.	This function confirms PubU based on UID and verifies the private key signature. If the verification passes and the timestamp in the signature is within the valid range, the counter c corresponding to the name PIN_x under the UID is set to 0.

**Table 8:** Stages involving cryptographic operations and the types and counts of operations.

Stage and Scenario	Steps	Number of Cryptographic Calculations
Scenario 1 of the Digital Identity Usage Stage	Step 1	1 E(2 Params) + 1 DS(1 Param)
	Step 4	1 E(2 Params) + 1 DS(4 Params)
Scenario 2 of the Digital Identity Usage Stage	Step 1	1 E(2 Params) + 1 DS(1 Param)
	Step 3	1 E(3 Params) + 1 DS(1 Param)
	Step 5	1 E(3 Params) + 1 DS(2 Param)
	Step 7	1 E(2 Params) + 1 DS(4 Params)
Scenario 1 of the Digital Identity Update Stage	Step 1	1 DS(4 Params)
	Step 2	1 E(1 Param) + 1 DS(3 Params)
	Step 3	1 E(5 Params) + 1 DS(1 Param)
	Step 4	1 E(6 Params) + 1 DS(1 Param)
	Step 6	1 E(6 Params) + 1 DS(1 Param)
	Step 7	1 E(6 Params) + 1 DS(1 Param)
	Step 8	1 DS(5 Params)
	Step 10	1 E(6 Params) + 1 DS(1 Param)

E represents encryption operations, DS represents digital signature operations, and Param(s) represents the number of parameters operated on.

## Data Availability

Data are contained within the article.
